# Photo Capture of Water by Single Crystals of a Nonporous Metal–Organic Material

**DOI:** 10.1021/jacs.6c01019

**Published:** 2026-03-30

**Authors:** Nevindee A. Samararathne, Davide M. Proserpio, Eric Reinheimer, Farshid Effaty, Tamador Alkhidir, Sharmarke Mohamed, Leonard R. MacGillivray

**Affiliations:** † Department of Chemistry, 166964 University of Iowa, Iowa City, Iowa 52242, United States; ‡ Departement de Chimie, 98629 Université de Sherbrooke, 2500 Bd de l’Université, Sherbrooke, Quebec J1K 2R1, Canada; § Università degli Studi di Milano, Dipartimento di Chimica, Via C. Golgi 19, 20133 Milano, Italy; ∥ Rigaku Americas Corporation, 9009 New Trails Drive, The Woodlands, Texas 77381, United States; ⊥ Institut Courtois, Université de Montréal, 1375 Ave. Thérèse-Lavoie-Roux, Montréal, Quebec H2V 0B3, Canada; # Department of Chemistry, Green Chemistry and Materials Modelling Laboratory, Khalifa University of Science and Technology, P.O. Box 127788, Abu Dhabi, United Arab Emirates; ○ Center for Catalysis and Separations, Khalifa University of Science and Technology, P.O. Box 127788, Abu Dhabi, United Arab Emirates

## Abstract

A nonporous metal–organic material (MOM) captures water from the atmosphere under ambient conditions upon exposure to light. The water capture is achieved via a single-crystal-to-single-crystal [2 + 2] photocycloaddition. The MOM adopts an unusual condensed version of the primitive cubic (**pcu**) topology. The light generates isolated cavities within the crystal lattice that capture the water with overall retention of the **pcu** topology. The work underscores how MOMs can be advanced for controlled water capture with implications for sustainable water resource development, exemplifying how nonporous crystalline materials can be developed to capture small molecules from the atmosphere in the presence of light.

## Graphical abstract

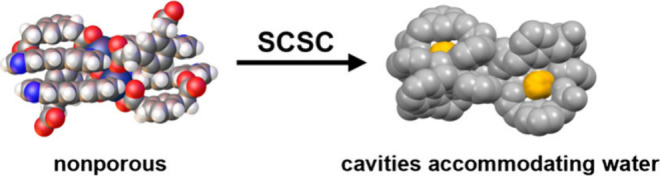

Materials that capture water are crucial to combat global water scarcity.^1–5^ According to the United Nations, water stress will affect nearly 5 billion people by 2050.^5^ Solutions to capture, store, and utilize water resources will support environmental sustainability and benefit regions with limited access to clean water.^6–10^ Molecular crystalline materials are promising for water capture with metal–organic frameworks (MOFs) being a focus.^11–13^ MOFs offer opportunities to control framework topologies to generate well-defined and accessible porous structures for guest uptake and release.^14–17^ The organic linkers of MOFs also allow for properties associated with smart materials (e.g., chemical responsiveness) to be integrated into a solid-state design.^18–20^ Generally, the structures of MOFs for water capture are predicated on offering accessible, open, and permanent pores to allow for entry, capture, and release.^2,21^

Herein, we describe a metal–organic material (MOM) with a topology based on a condensed primitive cubic (**pcu**) lattice that captures atmospheric water upon UV irradiation. Specifically, we show the pillars of Cd­(**3,3′-BPE**)­(**1,3-PDAc**) (where **3,3′-BPE** = *trans*-1,2-bis­(3-pyridyl)­ethylene; **1,3-PDAc** = 1,3-phenylenediacetate) to undergo a [2 + 2] photodimerization that is accompanied by spontaneous capture of water from the atmosphere in photogenerated Cd_2_(**3,3′-TPCB**)­(**1,3-PDAc**)_2_ (where **3,3′-TPCB** = *rctt*-tetrakis­(3-pyridyl)­cyclobutane) (Scheme 1a). Remarkably, the parent crystalline solid Cd­(**3,3′-BPE**)­(**1,3-PDAc**) is completely devoid of cavities for water capture. However, UV-triggered cycloaddition creates isolated, well-defined cavities that spontaneously trap atmospheric water as discrete, hydrogen-bonded dimers within the crystal. Molecular dynamics (MD) simulations show that these confined dimers persist over time, remaining hydrogen-bonded to one another while forming dynamic O–H···O contacts with the framework. Density functional theory (DFT) calculations demonstrate that water uptake into the photogenerated cavities is thermodynamically favorable.

**Scheme 1:**
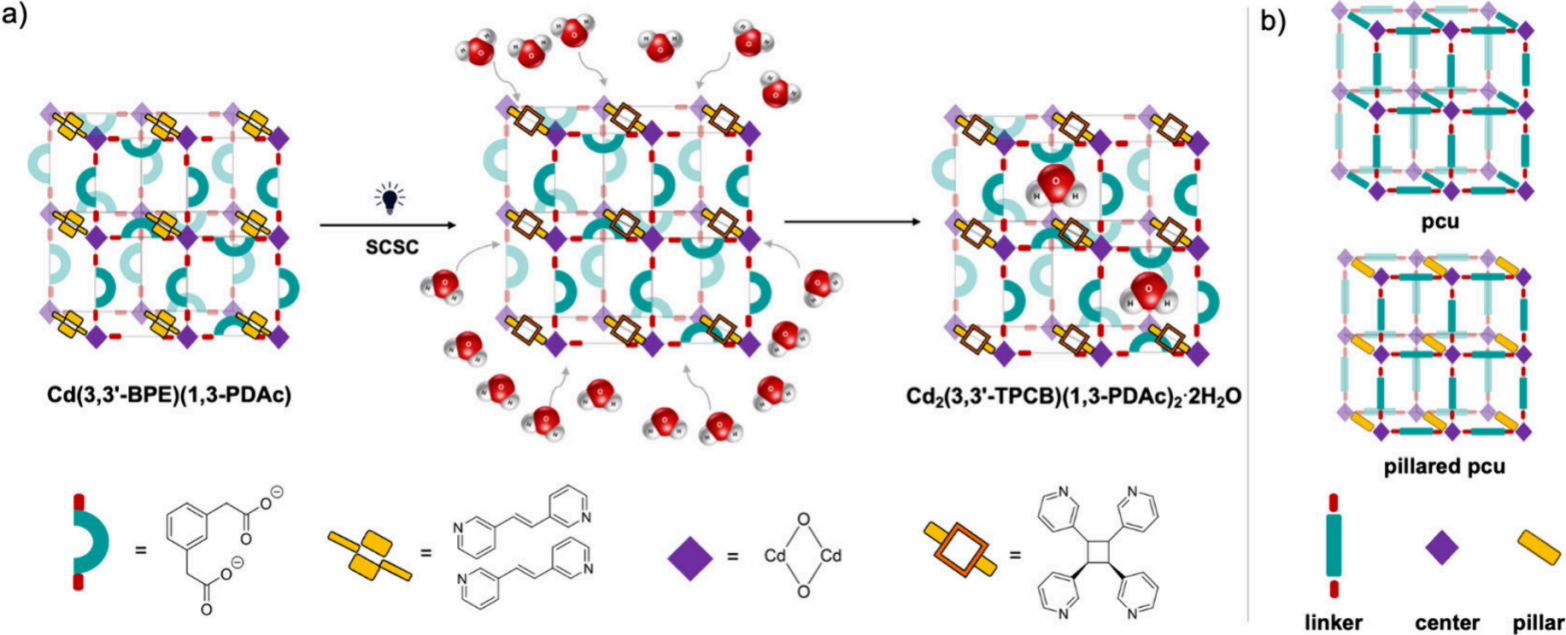
(a) Water Uptake of Cd­(**3,3′-BPE**)­(**1,3-PDAc**) Using UV Light to Form Cd_2_(**3,3′-TPCB**)­(**1,3-PDAc**)_2_ and (b) Primitive Cubic (**pcu**) and Pillared **pcu** Topologies

The **pcu** framework is an important MOF topology.^22,23^ The framework is composed of linkers disposed octahedrally in an ideally cubic lattice to generate an open 3D structure (Scheme 1b). An alternative construction of a **pcu** topology involves linking 2D square grids with pillars.^24–26^ Zaworotko reported host–guest properties of the well-known pillared **pcu** framework *SIFSIX*, which was later exploited by Kitagawa for gas uptake.^27–29^ Yaghi reported prototypal MOF-5 based on a **pcu** topology with all linkers of identical composition.^30,31^

We determined here that UV light triggers water capture into the condensed or nonporous (*cf*. porous) 3D double-pillared MOM Cd­(**3,3′-BPE**)­(**1,3-PDAc**). The uptake occurs in a single crystal-to-single crystal (SCSC) transformation with the **pcu** topology being retained while featuring the generation of isolated water-filled cavities following application of the light.

When a Teflon-lined autoclave was loaded with Cd­(NO_3_)_2_·4H_2_O (0.5 mmol) and **1,3-H**_**2**_ **PDAc** (0.5 mmol), **3,3′-BPE** (0.5 mmol), and H_2_O (25 mL), sealed, and heated to 175 °C for 3 days, light-yellow crystals of Cd­(**3,3′-BPE**)­(**1,3-PDAc**) formed. The composition of Cd­(**3,3′-BPE**)­(**1,3-PDAc**) was confirmed by ^1^H NMR spectroscopy and X-ray diffraction.

Crystals of Cd­(**3,3′-BPE**)­(**1,3-PDAc**) adopt the monoclinic space group *P*2_1_/*n* (Figure 1). Each Cd­(II) ion sits in an approximate pentagonal bipyramidal geometry. Two pyridyl N atoms of **3,3′-BPE** coordinate axially (Figure 1a) while the remainder of the coordination environment consists of O atoms from two chelating carboxylate groups and a bridging O atom of a carboxylate to generate Cd­(II) dimeric units (Cd···Cd 3.76 Å). The chelation consists of pairs of near symmetrically (Cd···O 2.415(3) and 2.522(3) Å) and unsymmetrically (Cd···O 2.265(3) and 2.609(3) Å) coordinated O atoms. The bridging methylenes are disordered over two sites (site occupancies: 0.63/0.37). The bifunctional nature of the **1,3-PDAc** ions means that the Cd­(II) dimers are bridged as rhombus grids within the crystallographic *ac*-plane (lengths: 11.46 Å; angles 63.2°, 116.8°) (Figure 1b).

**Figure 1:**
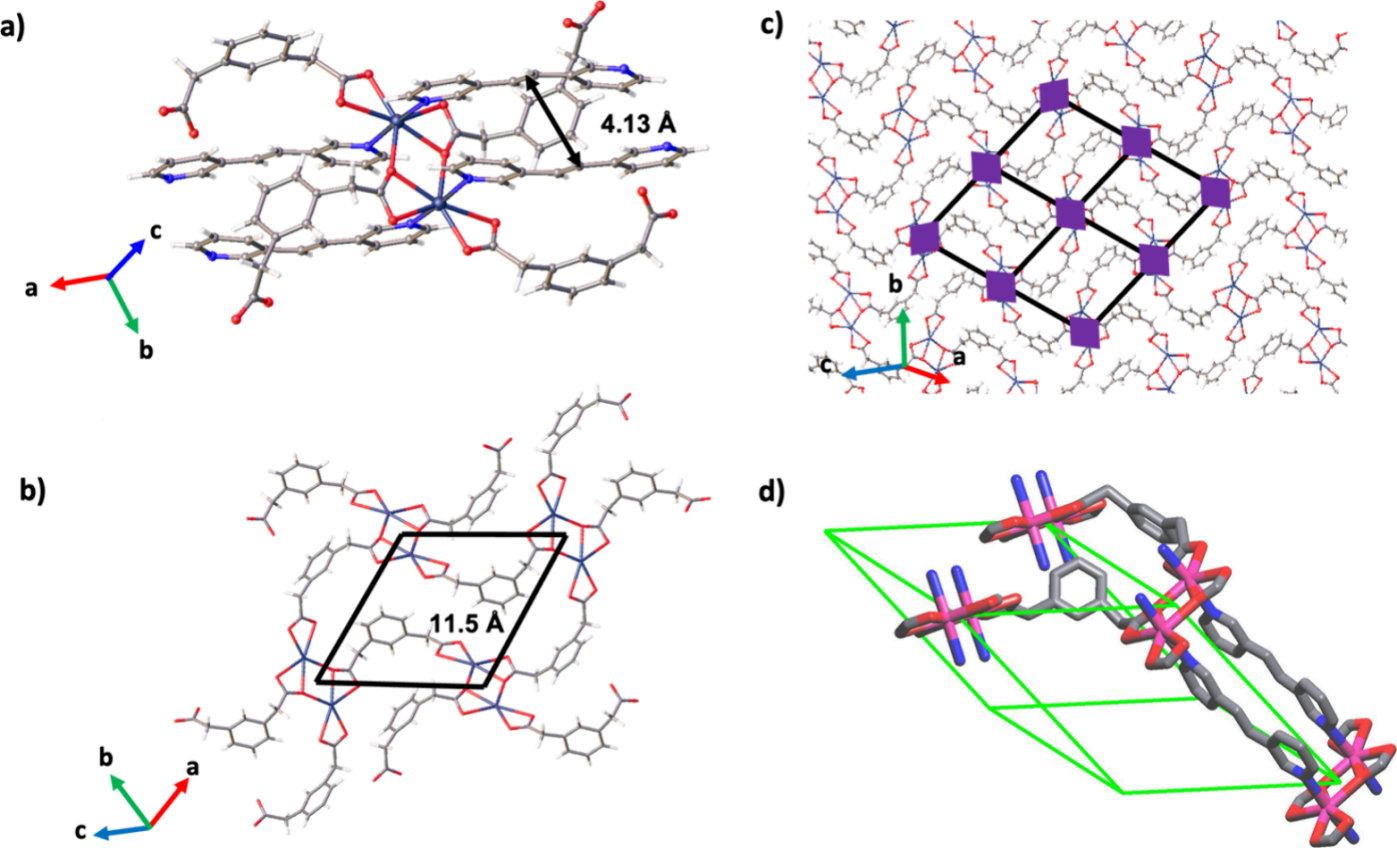
X-ray Cd­(**3,3′-BPE**)­(**1,3-PDAc**): (a) Cd­(II) dimers, (b) rhombus structure, (c) 2D grid, and (d) cuboidal tile (green) of **pcu** underlying net showing one double-bridged edge (colors: Cd = dark blue or pink; O = red; N = blue; C = gray).

The resulting rhombus grids of Cd­(**3,3′-BPE**)­(**1,3-PDAc**) lack cavities owing to the undulating nature of the U-shaped dicarboxylates, which results in space being filled and fully occupied (Figure 1c). The Cd­(II) dimers are linked by axially coordinated stacked **3,3′-BPE** molecules. The dimers act as double pillars (pillar grid-to-grid: 12.5 Å) to give a 3D and completely nonporous (*ca*. 8.5 Å^3^/Cd-dimer) framework based on a pillared **pcu** topology. **3,3′-BPE** is a nonlinear linker, which means adjacent grids are offset (angle: 15.3°).

The Cd­(II) dimeric units are positioned at the vertices of the **pcu** framework, while pairs of **3,3′-BPE** linkers are distributed parallel along four edges of the cuboidal tile of **pcu** (Figure 1d). The MOM completely lacks any void space. The lack of cavities and pores throughout Cd­(**3,3′-BPE**)­(**1,3-PDAc**) contrasts with typical **pcu** MOFs that are open and, in some cases, may interpenetrate to fill space.^32^ The framework is, therefore, essentially a “condensed” or a “collapsed” **pcu** structure.

The stacked double pillars of **3,3′-BPE** are organized parallel with the C=C bonds separated by 4.13 Å (Figure 1a). The geometry makes the C=C bonds suitable to undergo a [2 + 2] photodimerization.^33^ When Cd­(**3,3′-BPE**)­(**1,3-PDAc**) was subjected to UV radiation (UV LED 365 nm, 6 h), **3,3′-BPE** underwent a photocycloaddition to form **3,3′-TPCB** stereospecifically and in quantitative yield. The cycloaddition was accompanied by the appearance of a cyclobutane peak (4.69 ppm) in the ^1^H NMR spectrum and the complete disappearance of the olefinic peak (7.39 ppm). Optical microscopy revealed the reacting single crystals remained intact (see Figure S12). Irradiation times shorter and longer than 6 h invariably resulted in lower yields and cracking of the crystals, respectively.

An X-ray structure investigation of the reacted single crystals confirmed generation of **3,3′-TPCB** to give Cd_2_(**3,3′-TPCB**)­(**1,3-PDAc**)_2_ (Figure 2). In the solid, the **pcu** topology remained intact (Figure 2a); however, the grids composed of the **1,3-PDAc** ions converted from rhombus to rhomboidal with different edge lengths (lengths: 11.71 Å, 11.39 Å; angles: 62.0°, 118.0°) (Figure 2b). The distance between Cd atoms of the elongated dimers increased from 3.77 to 3.89 Å with the generation of the rhomboidal grids. There was also a significant disruption to the coordination bonds.^34,35^ A Cd–O bond of an unsymmetrically chelated carboxylate group of a **1,3-PDAc** ion became disordered (occupancies: O8A 0.53, O8B 0.47) and underwent weakening to breakage (Cd···O8 2.55(3)/2.94(4) Å) (Figure 2c). The changes to the Cd–O bonds were accompanied by the coordination of the Cd­(II) ions undergoing a change from a pentagonal bipyramid to a highly distorted octahedron. The symmetry of the single crystals also changed from monoclinic to triclinic with an increase of approximately 3% in the unit cell volume.

**Figure 2:**
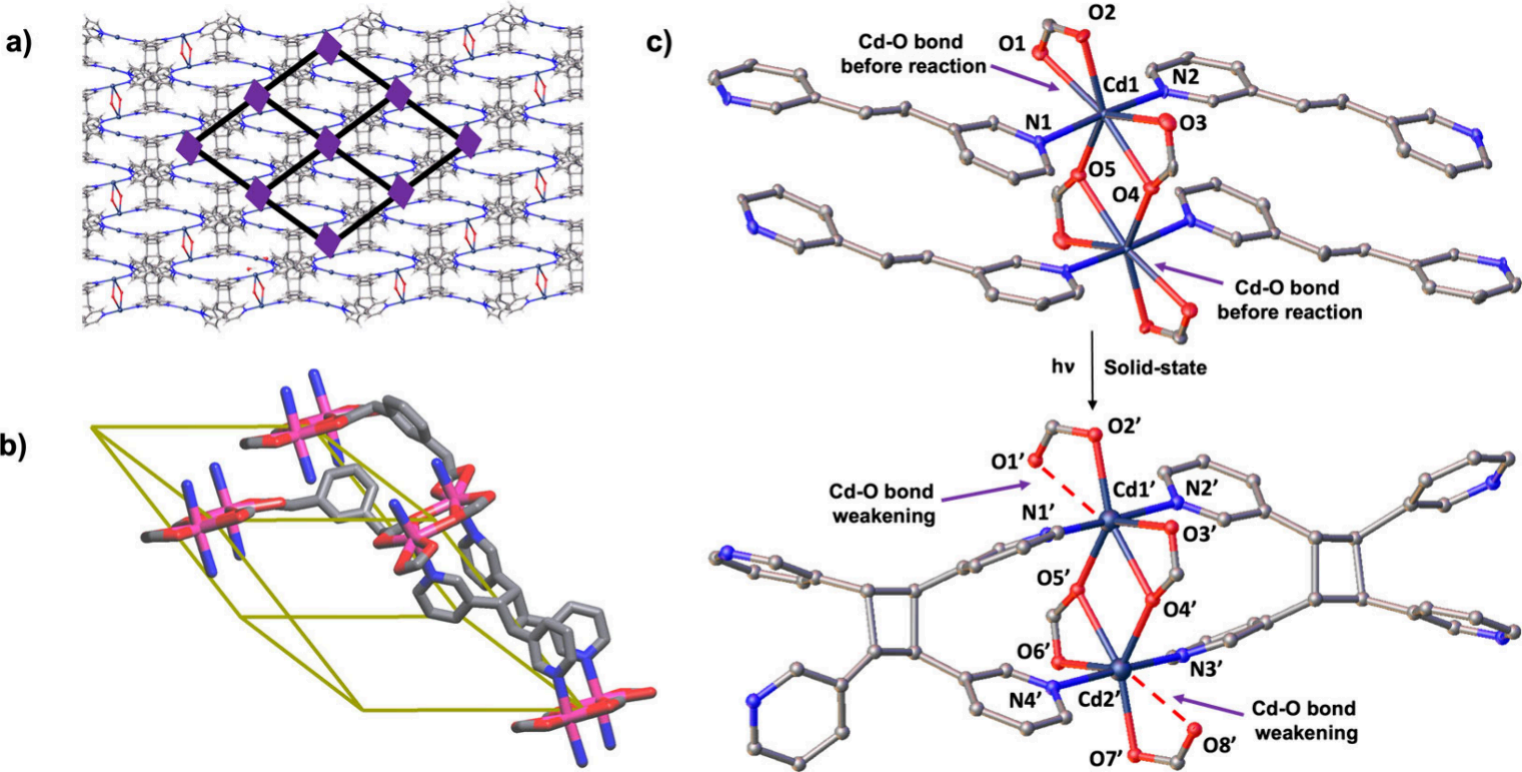
X-ray structure showing retention of **pcu** in Cd2­(**3,3′-TPCB**)­(**1,3-PDAc**)­2: (a) 2D grid with **3,3′-TPCB**, (b) uninodal topology of **pcu**, and (c) change of Cd­(II) coordination to distorted octahedron.

Remarkably, the photoreaction to generate **3,3′-TPCB** resulted in the formation of isolated cavities within the single crystals (Figure 3). The cavities lie adjacent to the Cd­(II) ions with the inner surfaces being lined by O atoms of the **1,3-PDAc** linkers and aromatic rings of **3,3′-TPCB** (66 Å^3^/unit cell) (Figure 3a, orange space-filling).

**Figure 3:**
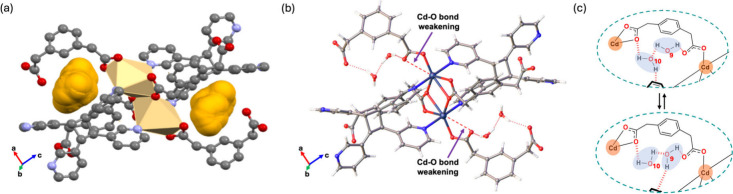
Cavities in Cd_2_(**3,3′-TPCB**)­(**1,3-PDAc**)_2_: (a) voids (yellow), (b) included water dimers, and (c) hydrogen bonding of dimers.

A closer inspection of the X-ray data reveals that the cavities are occupied by water molecules. Two partially occupied waters [occupancies: O(9) 0.36(3), O(10) 0.77(3)] assemble in each cavity (mass of waters = 2.09%). The assembly process is consistent with cavities able to accommodate two waters that participate in hydrogen-bonded dimers (O(9)···O(10) 2.64(4) Å) (i.e., water content up to 3.70%) (Figure 3b), wherein each dimer participates in two O–H···O hydrogen bonds (2.64(5) Å and 2.82(2) Å) to two **1,3-PDAc** ions. Waters O(10) and O(9) interact with coordinated and uncoordinated O atoms, respectively, with the latter originating from the weakened Cd–O bond. The remaining −OH groups participate in a series of O–H···π hydrogen bonds (O(10)···C(24) 3.47(2) Å) to a pyridyl ring. A hydrogen bonding pattern can be deduced that involves interconversion of the directionality of hydrogen bonding in each cavity (Figure 3c).

The presence of water in the photogenerated cavities is supported by thermogravimetric analysis data, which show a gradual mass loss from the powder (4.00%). The loss (see Figure S13) begins slightly above room temperature and is continuous up to 120 °C. The loss corresponds to the two waters in the cavities of the powder. A mass spectrum performed in lock mass or selected ion mode (18 *m*/*z*) shows the loss corresponds to water over both temperature and time (see Figure S14). The water molecules are likely loosely held in the individual cavities of the solid, which is corroborated by the combined crystallographic, mass spectrometric, and thermal data. The application of UV light, moreover, induced an expansion of the crystal lattice in a process akin to breathing “inhalation”,^36^ while preserving the underlining **pcu** topology. The expansion corresponds to significant void space to capture the water from the atmosphere (see Figure S17). We are unaware of a case wherein a photochemical reaction of a nonporous MOM generates isolated cavities; here, the formation of the cavities is accompanied by the uptake of water within the single crystals.

A dynamic vapor sorption (DVS) analysis was performed on a single crystalline sample of Cd­(**3,3′BPE**)­(**1,3-PDAc**) and Cd_2_(**3,3′-TPCB**)­(**1,3-PDAc**)_2_ before and after photoreaction, respectively. A full sorption–desorption cycle was employed from 0% RH up to 95% RH (see Figure S15). While the DVS profile for unreacted Cd­(**3,3′BPE**)­(**1,3-PDAc**) did not show a mass change upon humidity exposure, the profile for photoreacted Cd_2_(**3,3′-TPCB**)­(**1,3-PDAc**)_2_ exhibited an *in situ* mass increase (2.31%) that is consistent with the single crystal X-ray data. The isotherm also displayed hysteresis, consistent with the presence of a strong hydrogen bonding network during water uptake.^37,38^ The DVS data suggest Cd_2_(**3,3′-TPCB**)­(**1,3-PDAc**)_2_ is reusable for atmospheric water harvesting.

The **pcu** topology is reported in MOFs with water capture as a focus.^39,40^ Derivatization of MOF-5 allows for water uptake through steric shielding of metal centers to circumvent pore degradation.^31^ A pillared **pcu** framework has also been reported to undergo a porous-to-nonporous **pcu**-to-**sql** (square grid) transformation triggered by humidity.^27^ The designs of both **pcu** frameworks were predicated on the presence of open frameworks for water uptake. Weakening and breakage of a Zn–O bond in MOF-5 has notably been reported for water uptake.^31^ Vittal has also described an open **pcu** with pillars that undergo a SCSC photoreaction.^32^ In contrast, a feature of pillared Cd­(**3,3′BPE**)­(**1,3-PDAc**) is that the **pcu** framework is completely nonporous yet the material uptakes water. The photodimerization creates the necessary water-accessible space in the single crystals to allow for uptake from the atmosphere. The transport of the water into the crystals is likely assisted by combined hydrogen bonding and van der Waals cooperativity^41–45^ and accompanied by Cd–O bond weakening and breakage.

Computational modeling corroborates the experimentally observed water capture. Periodic DFT calculations yield an adsorption energy of −1.77 eV for two waters occupied as Cd_2_(**3,3′-TPCB**)­(**1,3-PDAc**)_2_·2­(**H**_**2**_ **O**) (−0.89 eV per H_2_O). Water uptake into the photogenerated cavities is thermodynamically favorable. Comparable dispersion-corrected DFT studies report significantly weaker binding for water in related MOFs with per-water adsorption energies of ca. −0.15 to −0.35 eV in MOF-5 and ca. −0.65 to −0.70 eV at open metal sites in MOF-74.^31,46^ The adsorption energy for Cd_2_(**3,3′-TPCB**)­(**1,3-PDAc**)_2_·2­(**H**_**2**_ **O**) lies at the upper end of the reported DFT range, which reflects strong cooperativity in intermolecular forces that stabilize water in the cavities.

Geometry optimization of Cd_2_(**3,3′-TPCB**)­(**1,3-PDAc**)_2_·2­(**H**_**2**_ **O**) (Figure 4) shows an equilibrium structure wherein two symmetry-unique waters form hydrogen-bonded dimers and engage in O–H···O interactions with the **1,3-PDAc** O atoms (Figure 4a). Electron-density difference maps reveal charge accumulation on water and framework O atoms and charge depletion at the water H atoms, indicating polarization and stabilization through cooperative intermolecular interactions (Figure 4b). MD simulations at 298 K provide further evidence that water dimers remain intact under thermal fluctuations with a narrow O···O distance distribution centered at 3.2 to 3.3 Å and H_2_O···O–Cd separations fluctuating at approximately 3.1 to 3.6 Å (Figure 4c). Collectively, the results establish a persistent yet dynamic hydrogen-bonding arrangement that confines water within isolated cavities of Cd_2_(**3,3′-TPCB**)­(**1,3-PDAc**)_2_·2­(**H**_**2**_ **O**), complementing the crystallographic and sorption data.

**Figure 4:**
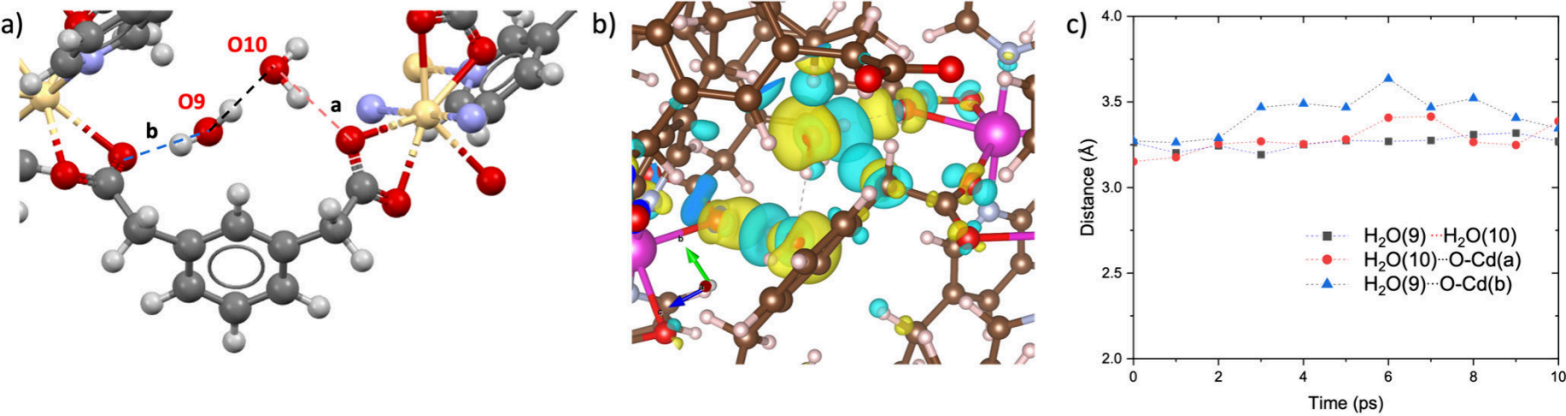
Computational analysis of water dimers in Cd_2_(**3,3′-TPCB**)­(**1,3-PDAc**)_2_: (a) hydrogen bonds between geometrically optimized waters, (b) electron-density map, and (c) evolution in intermolecular distances as a function of time during MD simulations.

To conclude, a photoreaction of the nonporous **pcu** framework Cd­(**3,3′BPE**) (**1,3-PDAc**) to the cavity-containing **pcu** Cd_2_(**3,3′-TPCB**)­(**1,3-PDAc**)_2_ via [2 + 2] photocycloaddition results in spontaneous capture of water from the atmosphere. We are expanding to linkers of increasing complexity with the idea that induced reactions and movements of organic linkers using light can trigger and achieve open spaces in MOMs. We believe our findings of light-induced water capture within a crystalline MOM demonstrate the potential of MOMs to advance developments in intelligent water harvesting technologies.^47,48^ Photostability and chemical durability tests are essential for practical applications.

## Supplementary Material





## Data Availability

All relevant data are available from the authors.

## Notes

Details of syntheses, NMR spectral data, PXRD diffractograms, topological representations, dynamic vapor sorption, thermal data, and computational modeling (PDF)
Molecular dynamics simulation showing water in cavities (Movie S1) (MP4)
