# Supplementary material for: Photo Capture of Water by Single Crystals of a Nonporous Metal–Organic Material
Source: J Am Chem Soc. 2026 Mar 30;148(17):17575–80. doi: 10.1021/jacs.6c01019 (PMC13154196; doi:10.1021/jacs.6c01019)
Supplement: Supplementary file 1 [file ja6c01019_si_001.pdf]

Photo Capture of Water by Single Crystals of a Nonporous Metal-Organic Material

Nevindee A. Samararathne,[1,2] Davide M. Proserpio,[3] Eric Reinheimer,[4] Farshid Effaty,[2,5] Tamador Alkhidir, [6,7] Sharmarke Mohamed [6,7] and Leonard R. MacGillivray[1,2*]

[1] Department of Chemistry, University of Iowa, Iowa City, IA, 52242, USA

[2] Departement de Chimie, Université de Sherbrooke, 2500 Bd de l'Université, Sherbrooke, QC, J1K 2R1, Canada

[3] Università degli Studi di Milano, Dipartimento di Chimica, Via C. Golgi 19, 20133 Milano, Italy

[4] Rigaku Americas Corporation, 9009 New Trails Drive, The Woodlands, TX, 77381, USA

[5] Institut Courtois, Université de Montréal, 1375 Ave. Thérèse-Lavoie-Roux, Montréal, QC H2V 0B3

[6] Department of Chemistry, Green Chemistry and Materials Modelling Laboratory, Khalifa University of Science and Technology, P.O. Box 127788, Abu Dhabi, United Arab Emirates

[7] Center for Catalysis and Separations, Khalifa University of Science and Technology, P.O. Box 127788, Abu Dhabi, United Arab Emirates

*E-mail: leonard.macgillivray@usherbrooke.ca

Supplementary Information

1. Synthesis of *trans*-1,2-bis(3-pyridyl)ethylene (**3,3'-BPE**)
2. Synthesis of Cd(**3,3'-BPE**)(**1,3-PDAc**)
3. ^{1}H NMR of Cd(**3,3'-BPE**)(**1,3-PDAc**)
4. ^{1}H NMR of Cd_2(**3,3'-TPCB**)(**1,3-PDAc**)$_2$
5. SCSC transformation of Cd(**3,3'-BPE**)(**1,3-PDAc**) to Cd_2(**3,3'-TPCB**)(**1,3-PDAc**)$_2$
6. Crystal structure determination
7. Crystallographic data of Cd(**3,3'-BPE**)(**1,3-PDAc**) and Cd_2(**3,3'-TPCB**)(**1,3-PDAc**)$_2 \cdot 1.13H_2O$
8. PXRD of Cd(**3,3'-BPE**)(**1,3-PDAc**)
9. PXRD of Cd_2(**3,3'-TPCB**)(**1,3-PDAc**)$_2$
10. **pcu** framework of Cd(**3,3'-BPE**)(**1,3-PDAc**)
11. **pcu** framework of Cd_2(**3,3'-TPCB**)(**1,3-PDAc**)$_2$
12. Optical microscopy images
13. TGA/DSC analysis of Cd_2(**3,3'-TPCB**)(**1,3-PDAc**)$_2$
14. TGA/DSC coupled with MS and IR
15. Dynamic Vapor Sorption (DVS) experiment
16. Comparison of bond lengths of the Cd-O coordination bond
17. Void volume calculation
18. Computational Modelling

1. Synthesis of *trans*-1,2-bis(3-pyridyl)ethylene (**3,3'-BPE**)

The alkene **3,3'-BPE** was synthesized as reported[1] using Pd-catalyzed Hiyama-Heck cross-coupling between 3-bromopyridine and triethoxyvinylsilane (2:1 molar ratio). Purification was achieved through flash chromatography (using SiO_2 and a mobile phase consisting of 10% MeOH/CH_2Cl_2), yielding **3,3'-BPE** as yellow crystals in a yield of 23%.

2. Synthesis of Cd(**3,3'-BPE**)(**1,3-PDAc**)

To a 50 mL Teflon-lined autoclave was loaded a mixture of $Cd(NO_3)_2.4H_2O$ (0.5 mmol), 1,3 Phenylenediacetic acid (**1,3-H$_2$PDAc**) (0.5 mmol), **3-3' BPE** (0.5 mmol) and H_2O (25 mL). The Teflon-lined autoclave was sealed and heated in an oven to 175 °C for 3 days and then cool to ambient temperature at a rate of 5 °C h^{-1} to form light-yellow crystals of Cd(**3,3'-BPE**)(**1,3-PDAc**) which were washed with ethanol and dried in air.[2]

3. ^{1}H NMR data for Cd(**3,3'-BPE**)(**1,3-PDAc**)

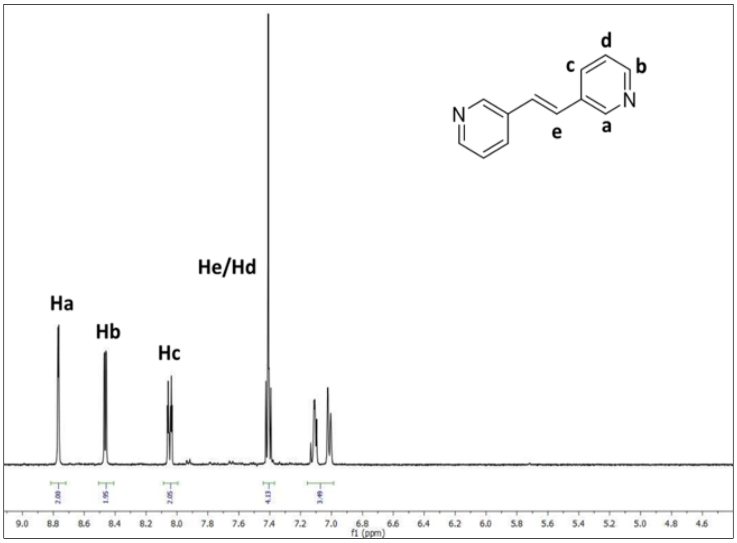

4. ^{1}H NMR data for Cd_2(**3,3'-TPCB**)(**1,3-PDAc**)$_2$

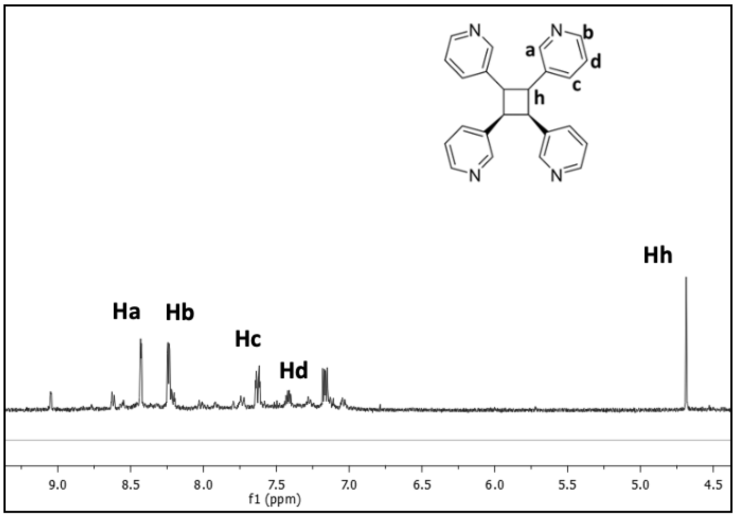

5. SCSC transformation of Cd(**3,3'-BPE**)(**1,3-PDAc**) to Cd$_2$(**3,3'-TPCB**)(**1,3-PDAc**)$_2$

The solid-state [2+2] photocycloaddition or photodimerization is a photochemical reaction in which two unsaturated molecules (i.e. alkenes) undergo a cycloaddition upon UV light exposure, forming a four-membered cyclobutane ring. In the solid state, this reaction occurs within a rigid crystal lattice, where the molecules are fixed in specific orientations. The reaction typically requires alignment of the reactants according to the Schmidt's topochemical criteria, as their proximity (approximately 4.2 Å) and parallel orientation in the crystal structure supports for effective overlap of orbitals. Solid-state [2+2] photodimerization is commonly used in materials science for the synthesis of photo-responsive materials and polymers.[3] While the reaction generally happens in a powdered solid, it is rare to observe the photodimerization occurring through a single-crystal-to-single-crystal (SCSC) transformation. In this work, SCSC [2+2] photodimerization was observed by irradiating single crystals with LED UV lights (wavelength peak at 365 nm) in a UV photoreactor (RT). Specifically, single crystals of Cd(**3,3'-BPE**)(**1,3-PDAc**) were subjected to UV light (LED UV panel with 441 lights) to generate Cd$_2$(**3,3'-TPCB**)(**1,3-PDAc**)$_2$. The progression of the reaction was monitored using NMR by irradiating the crystals in 1hr cycles. It was determined that 6hrs of irradiation resulted the fully conversion of Cd(**3,3'-BPE**)(**1,3-PDAc**) to Cd$_2$(**3,3'-TPCB**)(**1,3-PDAc**)$_2$. After 6hrs of irradiation single crystals retained the size and shape which was confirmed by optical microscopy. Further exposure caused the crystals to slowly turn opaque and to show signs of melting. NMRs shown below indicate partially converted material in 2hr and 4hr irradiation.

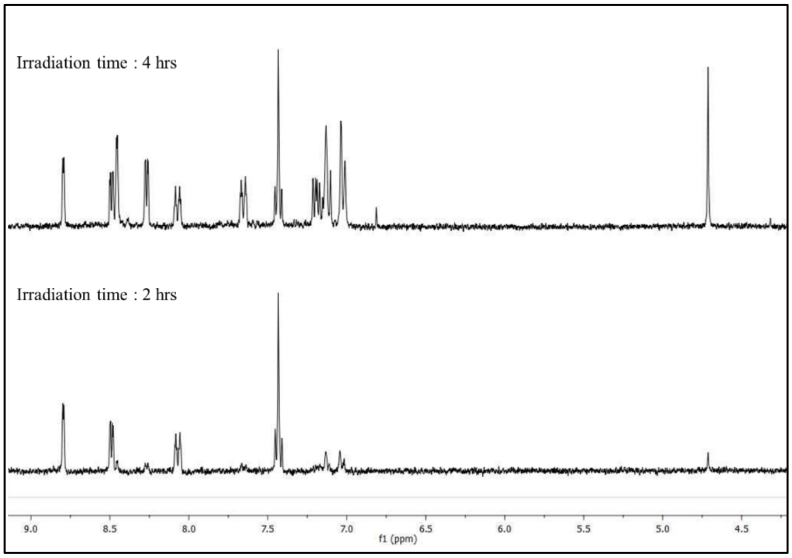

6. Crystal structure determination

Single-crystal X-ray diffraction data were collected on a Bruker D8 Venture Duo diffractometer using Mo Kα radiation (λ=0.71073 Å) with APEX II detector. Crystals were mounted in NVH oil on a Mitegen magnetic mount. Lorentz and polarization corrections with programs from the APEXII package were used for data reduction. Structure solution and refinement were completed using SHELXT3 and SHELXL4, respectively within the Olex2.5 graphical user interface. Non-hydrogen atoms were refined anisotropically. Hydrogen atoms were positioned geometrically and refined using a riding model.[4]

7. Crystallographic data for Cd(**3,3'-BPE**)(**1,3-PDAc**) and Cd_2(**3,3'-TPCB**)(**1,3-PDAc**)$_2$·$2H_2O$

Identification code	Cd(**3,3'-BPE**)(**1,3-PDAc**)	Cd_2(**3,3'-TPCB**)(**1,3-PDAc**)$_2$·1.13 H_2O
CCDC deposition number	2441351	2441352
Empirical formula	$C_{22}H_{18}CdN_2O_4$	$C_{44}H_{38.2}Cd_2N_4O_{9.1}$
Formular weight g/mol	486.79	1992.22
Temperature/K	100(2)	100(2)
Crystal system	Monoclinic	Triclinic
Space group	$P2_1/n$	P-1
a/Å	12.5430(9)	10.2641(16)
b/Å	12.0111(9)	10.4694(16)
c/Å	12.9583(10)	19.793(3)
α/°	90	86.596(4)
β/°	99.913(3)	84.454(4)
γ/°	90	70.096(4)
Volume/Å^3	1923.1(3)	1989.7(5)
Z	4	2
ρ_{calc} (g/cm^3)	1923.1(2)	1989.7(5)
R_1	0.0411	0.0880
WR_2	0.0834	0.2563
μ/mm^{-1}	1.681	1.659
F(000)	976	998
Crystal size/mm^3	0.069 x 0.117 x 0.158	0.045 x 0.107 x 0.200
Goodness-of-fit on F^2	1.076	1.049

8. PXRD diffractogram for Cd(**3,3'-BPE**)(**1,3-PDAc**)

Powder X-ray diffraction (PXRD) data were collected on a Bruker D8 Advance X-ray diffractometer using CuKα1 radiation (λ = 1.5418 Å) in the range 5–40° (scan type: coupled TwoTheta/Theta; scan mode: continuous PSD fast; step size: 0.019°) (40 kV and 30 mA).

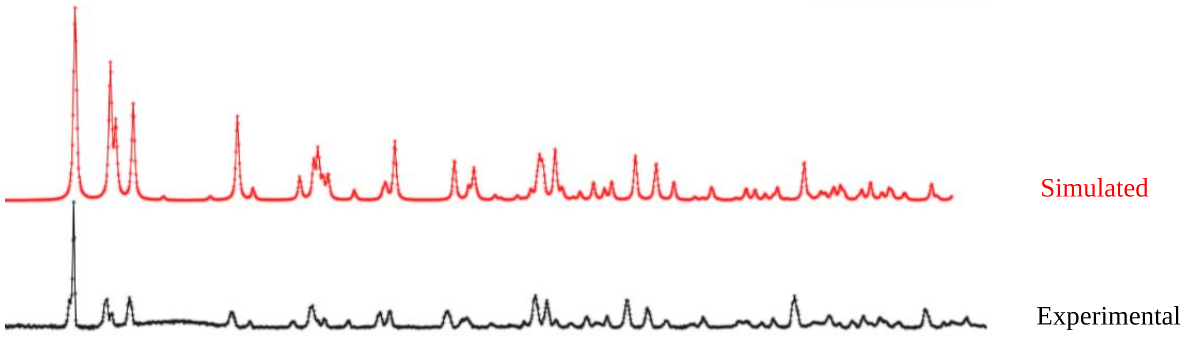

9. PXRD diffractogram of Cd_2(**3,3'-TPCB**)(**1,3-PDAc**)$_2$

Powder X-ray diffraction (PXRD) data were collected on a Bruker D8 Advance X-ray diffractometer using CuKα1 radiation (λ = 1.5418 Å) in the range 5–40° (scan type: coupled TwoTheta/Theta; scan mode: continuous PSD fast; step size: 0.019°) (40 kV and 30 mA).

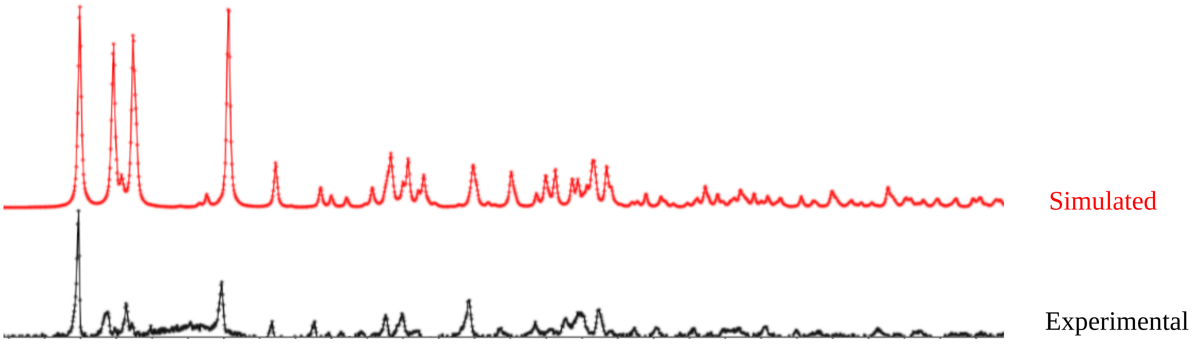

10. Representation of **pcu** framework derived from X-ray data of Cd(**3,3'-BPE**)(**1,3-PDAc**)

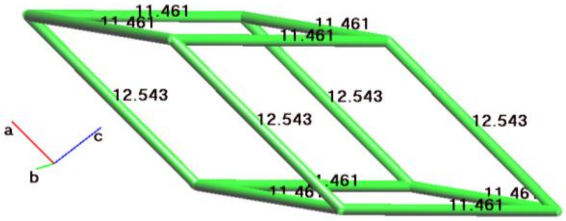

The analysis of the structures was done with ToposPro[5] and the underlying net were determined using the cluster simplification.[6] The observed **pcu** nets have 4 parallel edges double bridged by 3,3'-BPE that became a single bridge by 3,3'-TPCB upon the SCSC transformation.

11. Representation of **pcu** framework derived from X-ray data of Cd_2(**3,3'TPCB**)(**1,3-PDAc**)$_2$

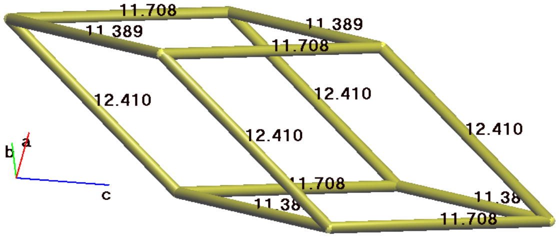

12. Optical Microscopy Images

Optical Microscopy was used to take images of the single crystals of Cd(**3,3'-BPE**)(**1,3-PDAc**) and Cd_2(**3,3'-TPCB**)(**1,3-PDAc**)$_2$, before and after the photoreaction, respectively.

Optical Microscopy image
Before the reaction

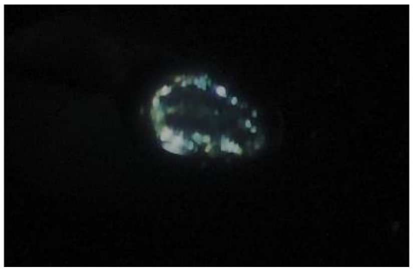

Optical Microscopy image
After the reaction (6h irradiation)

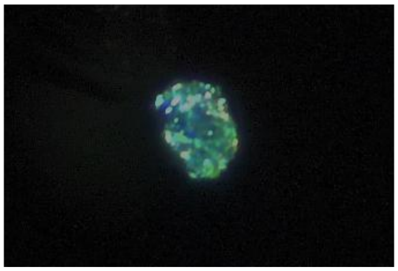

13. Thermal Gravimetric Analysis/Differential Scanning Calorimetry (TGA/DSC) analysis of Cd_2(**3,3'-TPCB**)(**1,3-PDAc**)$_2$

Thermal gravimetric analysis (TGA) and differential scanning calorimetry (DSC) were carried out simultaneously using SDT- Q600 V20.0 (TA instrument Delaware, USA). Indium-certified reference material was used in the calibration. About 3 mg of samples were weighed out and crippled in the alumina pan. The samples were analyzed at the heating rate of 5°C/min and were heated from 10°C to 300°C under a continuously purged dry nitrogen environment (flow rate 20 mL/min). TGA mass loss was calculated as 4.0% by using the inflection point of the curve.

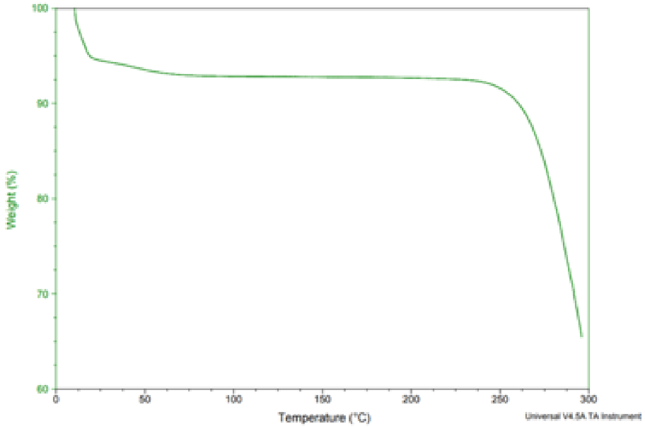

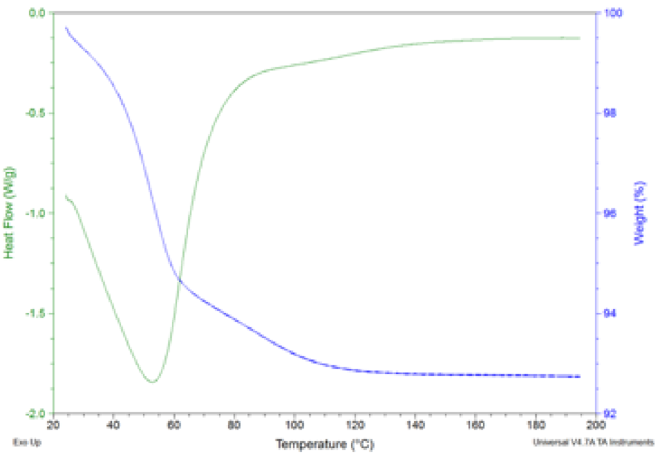

14. Thermal Gravimetric Analysis/Differential Scanning Calorimetry Coupled with Mass Spectrometry and Infrared Spectroscopy (TGA/DSC-MS-IR)

The thermal stability and decomposition behavior of the Cd_2(**3,3'-TPCB**)(**1,3-PDAc**)$_2$·$2H_2O$ were investigated using a combined Thermal Gravimetric Analysis (TGA) and Differential Scanning Calorimetry (DSC) system coupled with Mass Spectrometry (MS) and Infrared Spectroscopy (IR). The experiments were performed on a TA Instruments Discovery SDT 650 equipped with a high-precision balance and a temperature-controlled furnace. 11.9 mg of the sample was placed in an alumina crucible and heated from ambient temperature (ca. 25 °C) to 350 °C at a constant heating rate of 5 °C/min under a nitrogen atmosphere (flow rate: 100 mL/min). The TGA measured the mass loss as a function of temperature, while the DSC simultaneously recorded the heat flow associated with thermal transitions. The evolved gases from the TGA/DSC were directly transferred to a single quadrupole Thermostar TM200 via a heated capillary transfer line maintained at 200 °C to prevent condensation. The mass spectrometer was operated in selected ion monitoring (SIM) mode, with the mass channel locked at 18 m/z to specifically detect water (H_2O) removal from the Cd_2(**3,3'-TPCB**)(**1,3-PDAc**)$_2$·$2H_2O$. The TGA output was synchronized with the MS data acquisition to correlate mass loss events with the release of water. Detailed MS spectra, including ion intensity profiles as a function of temperature and time are plotted.

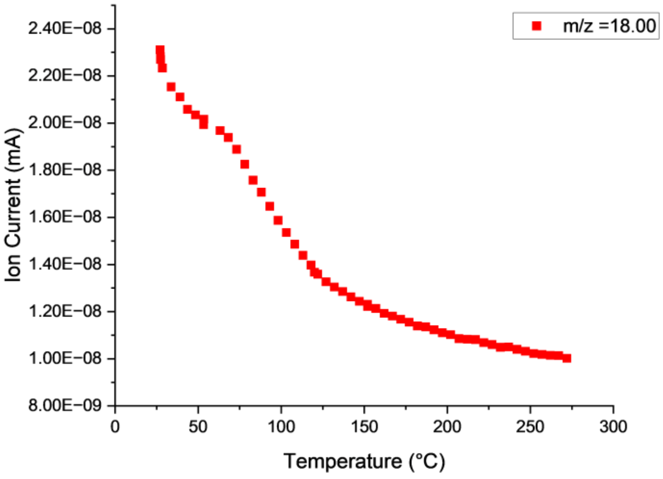

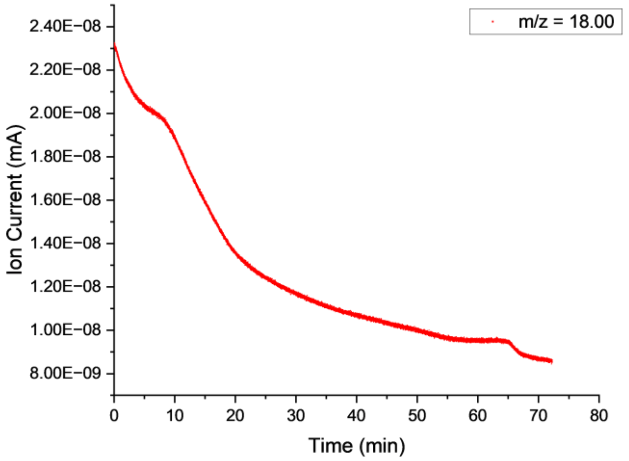

Overlayed IR Spectra

1-10 mins (27 °C – 37 °C)

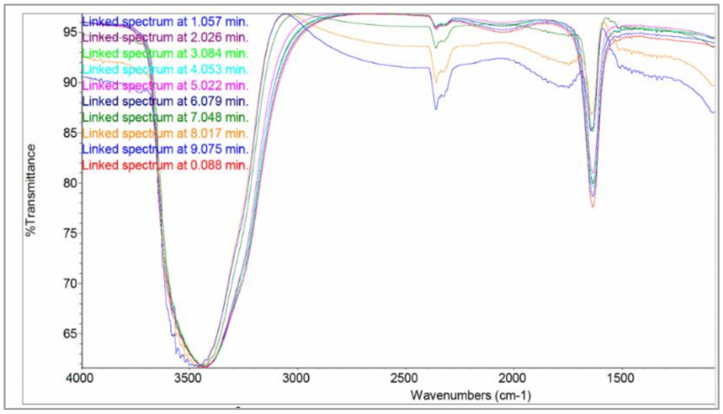

12-20 mins (39 °C – 47 °C)

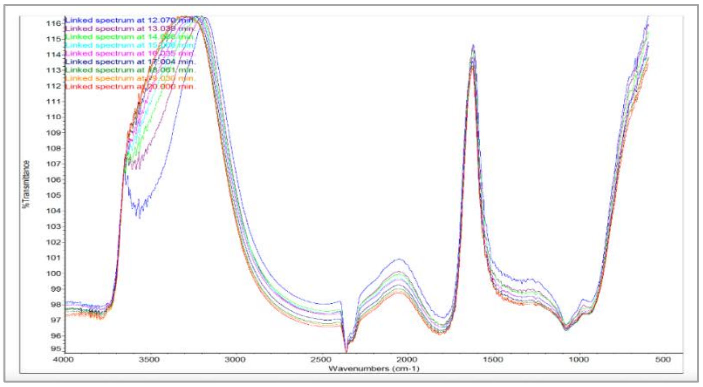

15. Dynamic Vapor Sorption (DVS)

To investigate the water uptake behavior of MOM, a Dynamic Vapor Sorption (DVS) analysis was performed under controlled relative humidity (RH) conditions at 25 °C. Prior to experiments, all samples were dried using a freeze-drying system for two days and the DVS measurements were conducted using the Surface Measurement Systems - Resolution instrument. A full sorption-desorption cycle was employed, starting from 0% RH to 95% RH in 5% increments, followed by desorption back to 0% RH. The temperature was maintained at 25 °C throughout the measurements. At each RH step, the sample was allowed to equilibrate to a dm/dt of 0.002% over a period ranging from 10 to 360 minutes.

After two days of freeze-drying in the dark, 4.0636 mg of Cd(**3-3'BPE**)(**1,3-PDAc**) was quickly transferred to the DVS chamber to minimize any photo-transformation. Another aliquot of Cd(**3-3'BPE**)(**1,3-PDAc**) (in crystalline powder form) was irradiated under UV light using a 370 nm Kessil LED lamp at a 5 cm distance, in a sealed glass vial filled with nitrogen gas for two days to induce photocycloaddition. The full conversion to the Cd_2(**3,3'-TPCB**)$(\textbf{1,3-PDAc})_2$ was confirmed by digesting the reacted sample in DMSO-d6 and subsequently analyzing it by ^1H NMR spectrum. A total of 5.0261 mg of photoreacted Cd_2(**3,3'-TPCB**)$(\textbf{1,3-PDAc})_2$ was transferred to the DVS chamber and dried for an additional 3 hours to ensure complete removal of moisture prior to measurement.

The DVS profile of Cd(**3-3'BPE**)(**1,3-PDAc**) showed no significant mass change (±0.1%) consistent with its initially nonporous nature. The photoreacted Cd_2(**3,3'-TPCB**)$(\textbf{1,3-PDAc})_2$ exhibited a mass gain of 2.312%, corresponding to approximately 1.25 equivalents of water absorbed per $C_{44}H_{36}Cd_2N_4O_8$ unit.

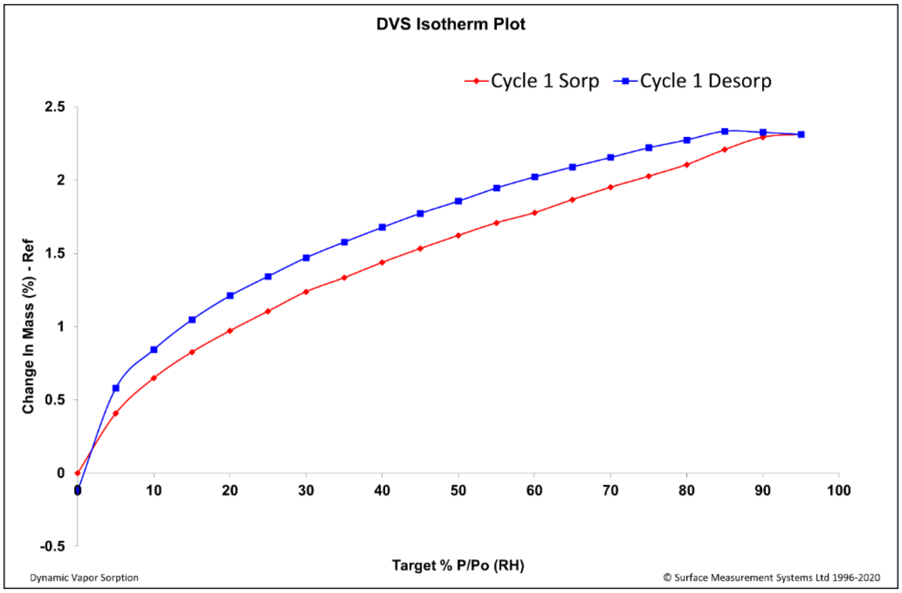

16. Comparison of bond lengths of the Cd-O coordination bonds

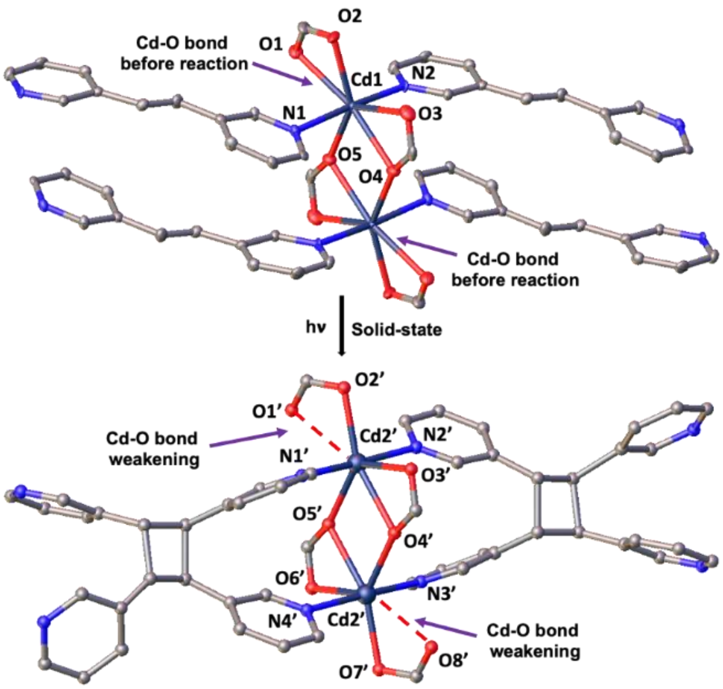

Cd-X	Before (X)
Cd1-O1	2.265(3)
Cd1-O2	2.609(3)
Cd1-O3	2.523(3)
Cd1-O4	2.414(3)
Cd1-O5	2.345(3)
Cd1-N1	2.358(4)
Cd1-N2	2.298(4)

Cd-X	After (X’)
Cd2’-O4	2.464(8)
Cd2’-O5	2.299(9)
Cd2’-O6	2.392(9)
Cd2’-O7’A	2.41(3)
Cd2’-O7’B	2.11(3)
Cd2’-O8’A	2.55(3)
Cd2’-O8’B	2.94(4)
Cd2’-N3	2.33(1)
Cd2’-N4	2.32(1)

17. Void volume calculation

Void volume calculations were carried out using Mercury (Cambridge Crystallographic Data Centre, CCDC). The crystallographic information file (CIF) of Cd(**3,3'-BPE**)(**1,3-PDAc**) was used as input for the analysis of the voids before the photoreaction. A probe radius of 1.0 Å and a grid spacing of 0.3 Å were employed for the calculations. The analysis revealed the structure before the photoreaction to be completely nonporous (viewed through crystallographic *b* axis).

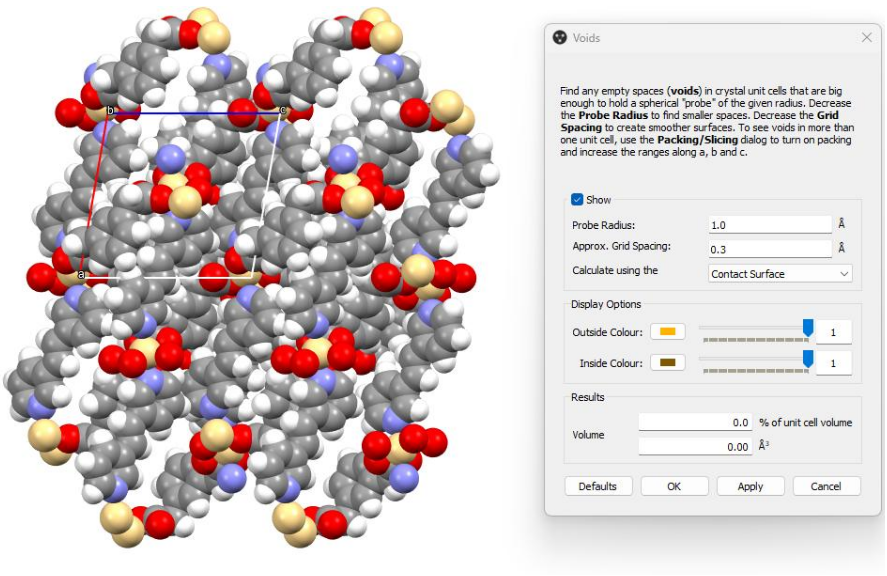

Water molecules present in the CIF file of photoreacted structure of Cd_2(**3,3'-TPCB**)(**1,3-PDAc**)$_2$ were removed and resulting CIF file of Cd_2(**3,3'-TPCB**)(**1,3-PDAc**)$_2$ was used as input for the analysis of the voids after the photoreaction. Void space within the unit cell was evaluated using the contact surface method. The void volume of the structure without water molecules was determined to be 66 Å^3**,** corresponding to 3.3% of the total unit cell volume. (viewed through crystallographic *b* axis – voids shown in yellow spheres)

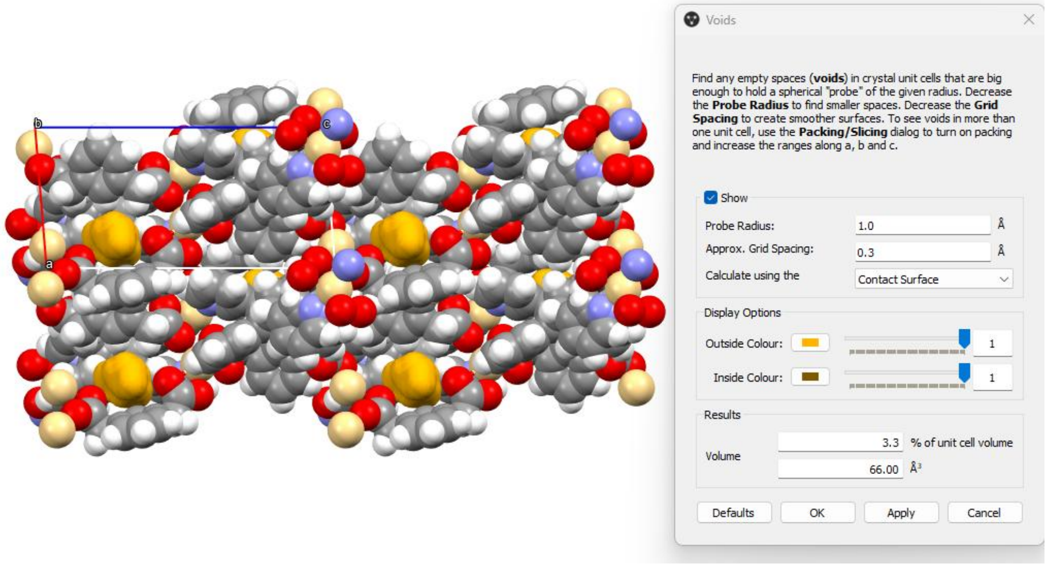

18. Computational Modelling

Density functional theory (DFT) calculations were performed using VASP,[7-9] which uses the projector-augmented wave (PAW)[10] method with plane-wave basis and PAW pseudo potentials. In all calculations, the PBE[11] generalized gradient approximation (GGA) was used for the exchange-correlational functional coupled with Grimme's D3 dispersion correction (PBE-D3) with Becke-Johnson damping function.[12] The initial structural model for Cd_2**(3,3′-TPCB)(1,3-PDAc)**$_2$·$2H_2O$ was taken from the single-crystal X-ray diffraction data. VASP input files were generated using the cif2cell program.[13] In all cases, a Γ-centered Monkhorst-Pack scheme was used to generate a tight K-point mesh with maximum K-point distance set to $2\pi \times 0.032$ Å^{-1}. The cut-off energy for the plane-wave basis set was set to 700 eV. Within each self-consistent field cycle, the convergence threshold was set at 1×10^{-5} eV and the geometry was considered converged when all forces were below 0.03 eV Å^{-1}.

Water adsorption energies were evaluated using three consistently treated equilibrium structures. The total energy of the fully optimized hydrated crystal, Cd_2**(3,3′-TPCB)(1,3-PDAc)**$_2$·$2H_2O$, was first obtained. The anhydrous reference structure was generated by removing the two water molecules from the hydrated structure and fully re-optimizing the resulting framework to obtain the equilibrium anhydrate. To explicitly retain water–water cooperativity, the water reference energy was computed by extracting the two water molecules in their optimized hydrated geometry, placing them together in a large vacuum simulation box (50 Å of vacuum), and computing the total energy of this water cluster using identical computational settings. The total adsorption energy for the two water molecules was defined as

$$E_{ads}^{(total)} = E_{hydrated} - (E_{anhydrous} + E_{water-cluster}^{(vac)})$$

and the per-water adsorption energy is defined as

$$E_{ads} = \frac{E_{ads}^{(total)}}{2}$$

Electron-density difference and electron localization analyses were performed using the same three equilibrium charge densities obtained from VASP calculations. Visualization was carried out using VESTA[14] to identify regions of charge accumulation and depletion associated with hydrogen bonding and framework polarization.

Molecular dynamics (MD) simulations were performed using the Forcite module in Materials Studio 2023.[15] A 3×3×3 supercell of the optimized **Cd_2(3,3′-TPCB)(1,3-PDAc)$_2$·$2H_2O$** structure was constructed. Simulations were carried out in the NVT ensemble at 298 K, using the Universal Force Field (UFF) with QEq charge equilibration and a Nosé thermostat. A time step of 1.0 fs was used. Electrostatic interactions were treated using the Ewald summation method with an accuracy 0.001 kcal mol^{-1}, while van der Waals interactions were computed using an atom-based summation method with a cubic spline truncation and a cutoff distance of 12.5 Å. MD trajectories were propagated in 10 ps segments, and distance metrics were extracted from a representative 10 ps trajectory that captures the relevant hydrogen-bond dynamics. "Medium" integration and convergence settings as implemented in Materials Studio 2023 were used throughout. Trajectories were analyzed for O···O and H_2O···O–Cd distance distributions and hydrogen-bond persistence, providing a dynamic complement to the static DFT results.

References

1. Quentin, J.; MacGillivray, L. R. Halogen versus Hydrogen Bonding in Binary Cocrystals: Novel Conformation a Coformer with [2+2] Photoreactivity of Criss-Crossed C=C Bonds. *ChemPhysChem* **2020**, *21*, 154–163.
2. Li, N.-Y.; Chen, J.-M.; Tang, X.-Y.; Zhang, G.-P.; Liu, D. Reversible Single-Crystal-to-Single-Crystal Conversion of a Photoreactive Coordination Network for Rewritable Optical Memory Storage. *Chem. Commun.* **2020**, *56*, 1984–1987.
3. Park, I.-H.; Lee, E.; Lee, S. S.; Vittal, J. J. Chemical Patterning in Single Crystals of Metal–Organic Frameworks by [2+2] Cycloaddition Reaction. *Angew. Chem., Int. Ed.* **2019**, *58*, 14860–14864.
4. Dolomanov, O. V.; Bourhis, L. J.; Gildea, R. J.; Howard, J. A. K.; Puschmann, H. OLEX2: A Complete Structure Solution, Refinement and Analysis Program. *J. Appl. Crystallogr.* **2009**, *42*, 339–341.
5. Blatov, V. A.; Shevchenko, A. P.; Proserpio, D. M. Applied Topological Analysis of Crystal Structures with the Program Package ToposPro. *Cryst. Growth Des.* **2014**, *14* (7), 3576–3586.
6. Bonneau, C.; O'Keeffe, M.; Proserpio, D. M.; Blatov, V. A.; Batten, S. R.; Bourne, S. A.; Lah, M. S.; Eon, J.-G.; Hyde, S. T.; Wiggin, S. B.; Öhrström, L. Deconstruction of Crystalline Networks into Underlying Nets: Relevance for Terminology Guidelines and Crystallographic Databases. *Cryst. Growth Des*. **2018**, *18* (6), 3411–3418.
7. Kresse, G.; Furthmüller, J., Efficiency of ab-initio total energy calculations for metals and semiconductors using a plane-wave basis set. *Computational Materials Science* **1996,** *6* (1), 15-50.
8. Kresse, G.; Furthmüller, J., Efficient iterative schemes for ab initio total-energy calculations using a plane-wave basis set. *Physical Review B* **1996,** *54* (16), 11169-11186.
9. Kresse, G.; Hafner, J., Ab initio molecular dynamics for liquid metals. *Physical Review B* **1993,** *47* (1), 558-561.
10. Kresse, G.; Joubert, D., From ultrasoft pseudopotentials to the projector augmented-wave method. *Physical Review B* **1999,** *59* (3), 1758-1775.
11. Perdew, J. P.; Burke, K.; Ernzerhof, M., Generalized gradient approximation made simple. *Phys. Rev. Lett.* **1996,** *77* (18), 3865-3868.
12. Grimme, S.; Ehrlich, S.; Goerigk, L., Effect of the damping function in dispersion corrected density functional theory. *J. Comput. Chem.* **2011,** *32* (7), 1456-1465.
13. Björkman, T., CIF2Cell: Generating geometries for electronic structure programs. *Computer Physics Communications* **2011,** *182* (5), 1183-1186.
14. Momma, K.; Izumi, F., VESTA 3 for three-dimensional visualization of crystal, volumetric and morphology data. *J. Appl. Crystallogr.* **2011,** *44* (6), 1272-1276.
15. BIOVIA, Dassault Systemes, Materials Studio, Version 2023, Dassault Systemes, San Diego, CA, USA.
